# Retention and Functional Effect of Adipose-Derived Stromal Cells Administered in Alginate Hydrogel in a Rat Model of Acute Myocardial Infarction

**DOI:** 10.1155/2018/7821461

**Published:** 2018-03-26

**Authors:** Bjarke Follin, Adam Ali Ghotbi, Andreas Ettrup Clemmensen, Simon Bentsen, Morten Juhl, Rebekka Harary Søndergaard, Lisbeth Drozd Lund, Mandana Haack-Sørensen, Philip Hasbak, Smadar Cohen, Rasmus Sejersten Ripa, Jens Kastrup, Annette Ekblond, Andreas Kjær

**Affiliations:** ^1^Cardiology Stem Cell Centre, the Heart Centre, Rigshospitalet, University of Copenhagen, Copenhagen, Denmark; ^2^Department of Clinical Physiology, Nuclear Imaging & PET and Cluster for Molecular Imaging, Department of Biomedical Sciences, Rigshospitalet, University of Copenhagen, Copenhagen, Denmark; ^3^Regenerative Medicine and Stem Cell Research Center and Avram and Stella Goldstein-Goren Department of Biotechnology Engineering, Ben-Gurion University of the Negev, Beer-Sheva, Israel

## Abstract

**Background:**

Cell therapy for heart disease has been proven safe and efficacious, despite poor cell retention in the injected area. Improving cell retention is hypothesized to increase the treatment effect. In the present study, human adipose-derived stromal cells (ASCs) were delivered in an in situ forming alginate hydrogel following acute myocardial infarction (AMI) in rats.

**Methods:**

ASCs were transduced with luciferase and tested for ASC phenotype. AMI was inducted in nude rats, with subsequent injection of saline (controls), 1 × 10^6^ ASCs in saline or 1 × 10^6^ ASCs in 1% (*w*/*v*) alginate hydrogel. ASCs were tracked by bioluminescence and functional measurements were assessed by magnetic resonance imaging (MRI) and ^82^rubidium positron emission tomography (PET).

**Results:**

ASCs in both saline and alginate hydrogel significantly increased the ejection fraction (7.2% and 7.8% at 14 days and 7.2% and 8.0% at 28 days, resp.). After 28 days, there was a tendency for decreased infarct area and increased perfusion, compared to controls. No significant differences were observed between ASCs in saline or alginate hydrogel, in terms of retention and functional salvage.

**Conclusion:**

ASCs improved the myocardial function after AMI, but administration in the alginate hydrogel did not further improve retention of the cells or myocardial function.

## 1. Introduction

The leading cause of death in the world is cardiovascular disease. Approximately one million people suffer myocardial infarction (MI) each year in the USA alone. Despite great improvements in treatment of these patients, an increasing group of patients still suffer from heart failure [1].

Within the past two decades, cellular therapy using mesenchymal stromal cells (MSCs) for treating heart disease has been proven safe and able to moderately enhance the function of the heart [2, 3]. MSCs can be obtained from almost every tissue in the body, but most studies have used adipose-derived MSCs (ASCs) or bone marrow MSCs [4]. The effect of the treatment is attributed to the paracrine potential of the MSCs [5, 6]. MSCs are capable of adapting their secretome to meet the needs of their surroundings. The abilities to modify immune responses, initiate angiogenesis, and provide trophic factors for damaged native cells make MSCs a perfect candidate for the treatment of myocardial infarction [6]. However, the administered MSCs are only present in the heart for a brief period of time, with evidence for 10% retention after 24 hours, and declining retention in the following weeks [7–9]. Increasing the retention of the transplanted cells using various methods has resulted in increased functional gain compared to the regular treatment in preclinical models [10]. Many of the applied methods to increase cell retention are more exploratory than directly translatable into the clinic. As such, the reliance on modification of the MSCs or the coinjection of complicated synthetic scaffolds makes the translation more difficult [11]. For clinical application, the cell product must be minimally modified from their original phenotype, and every modification to scaffolds or hydrogels makes the regulatory process much more difficult. As the most translatable choice for administration, we investigated an in situ formed alginate hydrogel formulation currently in clinical trials as monotherapy for acute myocardial infarction (AMI) [12–14]. The unique features of this hydrogel are due to the fabrication as a flowable cross-linked alginate network, which undergoes gelation into a hydrogel at the presence of the Ca^2+^ ion concentrations available at the infarcted heart [15].

We have previously demonstrated that ASCs maintained high viability during culture in this alginate hydrogel, and that ASCs modify their phenotype and exhibit increased paracrine immunomodulatory function while embedded in the alginate hydrogel [16, 17].

In the present study, we investigated whether injecting transduced ASCs in an in situ formed alginate hydrogel would increase the retention of the cells and improve myocardial function in rats after AMI.

To do so, we tracked the ASCs by bioluminescence (BLI), used cardiac magnetic resonance imaging (MRI) to assess heart volumes and pumping function, and used ^82^rubidium positron emission tomography/computed tomography (^82^Rb-PET/CT) to assess the perfusion of the heart. To the best of our knowledge, we are the first to apply ^82^Rb-PET/CT in a small animal model to assess the effect of treatment in myocardial infarction.

## 2. Methods

### 2.1. Experimental Design

**Figure 1:**
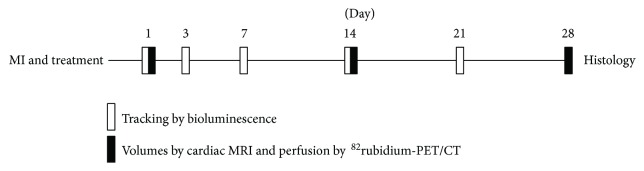
Experimental setup. Myocardial infarction was induced and rats were treated with either saline, ASCs in saline, or ASCs in alginate hydrogel on day 0. The ASCs were tracked at days 1, 3, 7, 14, and 21 after administration, with a subgroup being tracked 6 hours after injection. Functional measurements were obtained using cardiac magnetic resonance imaging (cardiac MRI) and rubidium-82 positron emission tomography/computer tomography (^82^Rb-PET/CT) at days 1, 14, and 28 postoperation.

Human ASCs were transduced to express luciferase and characterized *in vitro*. An experimental model for acute myocardial infarction (AMI) in rats was established with injection of saline (control), ASCs in saline, or ASCs in alginate hydrogel immediately following infarction. The functional measurements were obtained with cardiac magnetic resonance imaging (MRI) and ^82^rubidium-PET/CT (^82^Rb-PET/CT) on days 1, 14, and 28 after the operation. Cell tracking by bioluminescence imaging (BLI) was performed at days 1, 3, 7, 14, and 21 after treatment (Figure 1). Treatment was randomized, and outcome assessment was blinded.

### 2.2. Cell Culture

Lipoaspirate was harvested from two healthy human donors (females, aged 37 and 55 years). The use of the lipoaspirate was approved by the National Ethical Committee protocol number H-3-2009-119 and informed written consent was signed by the donors.

ASCs were isolated from the lipoaspirate, as described elsewhere [18]. Briefly, the lipoaspirate was washed twice with phosphate-buffered saline (PBS) (Gibco, Life Technologies) before being digested with 0.6 PZ U/ml collagenase NB 4 (Serva) dissolved in Hank's balanced salt solution (2 mmol/l Ca^2+^, Thermo Fisher Scientific) for 45–55 min, while incubated at 37°C at constant rotation. Collagenase was inactivated by addition of fetal bovine serum (FBS) medium (minimum essential medium alpha modulation (*α*MEM), 1% penicillin/streptomycin, and 10% irradiated FBS (all from Gibco, Thermo Fisher Scientific)). The suspension was centrifuged, and the mononuclear cells were counted on a NucleoCounter NC-100 (ChemoMetec), seeded in a density of 4.5 × 10^6^ cells/T75 flask (Thermo Fisher Scientific) in FBS medium, and incubated in standard conditions at 37°C, 5% CO_2_, and 21% O_2_ with humidified air. After three days of culture, the medium was removed, and the flasks were washed with PBS for the removal of nonadherent cells. Medium was changed twice a week, until confluence was reached and the cells were washed with PBS, detached with 3 ml TrypLE Select (Gibco, Life Technologies), and centrifuged at 300*g* for 5 min. After an additional culture passage, the ASCs were counted and frozen in liquid nitrogen in a density of 1 × 10^6^ cells/ml in 10% dimethylsulfoxide (WAK-Chemia Medical), 90% FBS.

### 2.3. Labeling

The L2T plasmid was supplied by Professor Gambhir, Stanford University, and a detailed description of the plasmid can be found elsewhere [19]. Briefly, the L2T plasmid consists of a firefly luciferase 2 sequence connected by a short linker to a td tomato sequence, with a ubiquitin promotor and zeocin resistance.

The L2T plasmid was propagated in bacteriophages. For packaging of the plasmid in lentiviral particles, HEK293T cells were seeded on day 1 in Dulbecco's modified Eagle's medium (DMEM), 10% fetal calf serum (AUS sourced, In Vitro Technologies), glutamine, and penicillin/streptomycin. On day 2, the HEK293T cells were transfected using CaPO_4_ transfection with the pFUG L2T vector and the two helper plasmids PDMG.2 and 8.91 (both from Didier Tromo Lab., Geneva), responsible for envelope and packaging, respectively. The transfection on day 2 was performed in 10% CO_2_, with conditions being changed to 3% CO_2_ until day 3. At the third day, the medium was changed and the incubation continued in 5% CO_2_. Medium was collected at day 5, filtered through a 0.45 *μ*l filter and concentrated 20 times using Vivaspin filter (cutoff 100.00 M_W_).

The transduction with the lentiviral particles was performed with 30.000 ASCs in each well in 6-well plates in FBS medium containing a titer of 25 mg virus per well and polybrene. After two days of quarantine, the transduced ASCs were transferred to T75 flasks and culture expanded in human platelet lysate (hPL) medium (*α*MEM, 1% penicillin/streptomycin, and 5% Cook Regentec Stemulate). The L2T-ASCs were expanded for one passage before being sorted by tdTomato expression, using a FACSJazz flow cytometry-activated cell sorter (BD Bioscience) at the Flow Cytometry Core Facility at the University of Copenhagen. The L2T-transduced ASCs were subsequently expanded for two passages, completing a total of 5 passages from their original isolation from the stromal vascular fraction, before being frozen, as described earlier [20].

Two to three weeks before the transduced ASCs were injected in the animals, they were thawed and cultured in hPL culture medium in T75 flasks at standard incubation conditions. Hence, the treated rats received L2T-ASCs in passage 7-8.

### 2.4. Characterization of L2T-Transduced ASCs

#### 2.4.1. Surface Marker Expression

The surface marker expression of the transduced ASCs was characterized by flow cytometry. The following antibodies (all purchased from BD Biosciences) were used: CD105-Brilliant Violet-421 (BV421) (clone 266), CD90-allophycocyanin (APC) (5E10), CD73-APC (AD2), CD13-phycoerythrin-cyanin 7 (PE-Cy7) (WM15), CD45-fluorescein isothiocyanate (FITC) (2D1), CD34-APC (581), and HLA-DR, DP, DQ-BV421 (Tu39). Fixable, viable stain 780 (FVS780) (cat. number 565388) was used to discriminate live from dead cells. Upon harvest, the cells were washed in PBS twice and stained with FVS780 for 10 min. Subsequently, the cells were washed in FACS PBS (Hospital Pharmacy) containing 1% ethylenediaminetetraacetic acid (EDTA) (Hospital Pharmacy) and 10% newborn calf serum (Gibco, Thermo Fisher Scientific) and stained for 30 min at room temperature, protected from light. Finally, the cells were washed in FACS PBS and resuspended in PBS. At least 4000 cells were measured on a Navios flow cytometer and analyzed using Kaluza software version 1.5a (both Beckman Coulter). Debris, doublets, and dead cells were excluded from the analysis.

#### 2.4.2. Triple Differentiation

The transduced ASCs were triple differentiated using StemPro differentiation kit (Gibco, Thermo Fisher Scientific) according to manufacturer's instructions. Adipogenic differentiation was verified by oil red O (Sigma-Aldrich) staining lipid droplets, osteogenic differentiation by alizarin red S for calcium deposits, and chondrogenic differentiation by alcian blue 8GX (Sigma-Aldrich) for glycosaminoglycans.

#### 2.4.3. *β*-Galactosidase Staining

To assess if the whole process of generating the transduced ASCs resulted in the cells being senescent, the endogenous *β*-galactosidase activity was investigated using the *β*-gal staining kit (Thermo Fisher Scientific) according to manufacturer's protocol. X-Gal was prepared fresh in N,N-dimethylformamide (Sigma-Aldrich Chemie GmbH) for staining solution. Cells were incubated for 24 hours at standard incubation conditions, and the staining was observed using an IX51 microscope (Olympus).

### 2.5. Alginate Hydrogel Casting

A 2.6% alginate solution (VLVG sodium alginate, NovaMatrix, FMC Biopolymers) was made in sterile water and partially cross-linked with a solution of 3% calcium gluconate (D-gluconic acid hemicalcium salt, Sigma-Aldrich) [21]. This mixture was subsequently diluted to a 1% *w*/*v* alginate and 0.3 calcium gluconate solution with sterile water. Prior to administration, a centrifuged cell pellet was resuspended in the alginate solution.

### 2.6. Animals

For this study, 97 athymic nude rats (Crl : Foxn1^rnu^) were included. The athymic nude rat was chosen based on evidence of lower levels of macrophage infiltration in the heart and improved long-term graft retention compared to other strains [9, 22]. All animal experiments were approved by the Danish Animal Experiments Inspectorate (permit number 2012-15-2934-00064 and 2016-15-0201-00920). The animals were housed in the core animal facilities at the University of Copenhagen, Denmark, with 12 : 12 hours light/dark cycle, at 21 ± 2°C, and access to water and rodent food ad libitum. The animals were acclimatized for at least one week before being included in the experiments. In order to test a cell product as close to the clinic as possible, we chose human ASCs as xenografts in athymic nude rats.

### 2.7. MI Induction and Treatment

The myocardial infarctions were induced as described elsewhere [23]. In brief, the animals were anesthetized in 3–5% sevoflurane (AbbVie, Denmark) in an induction chamber before being intubated with a 16 G Venflon catheter (Vasofix® Safety, Braun, Denmark) with blunt needle and ventilated with 6–8 ml air/ventilation, at a frequency 80–90 ventilations/min (UNO microventilator-03, Netherlands). The animals were injected subcutaneously with 0.05 mg/kg buprenorphine (Temgesic®, Indivior, UK) and 1 ml saline. Left-sided thoracotomy was performed at the 4th or 5th intercostal space. The pericardium was gently removed and the left anterior descending coronary artery (LAD) was permanently ligated caudal of its origin with a 6–0 polypropylene suture. Ischemia was confirmed visually by discoloring and dyskinesia of the myocardium. Following LAD ligation, 0.1 ml fluid was injected by two-three injections into the border zone of the discolored area. Animals received either saline 1 × 10^6^ ASCs in saline or 1 × 10^6^ ASCs in alginate hydrogel. Sham animals underwent the same procedure, with ligation in the myocardium instead of the LAD. The thorax, muscle layers, and skin were closed with 4-0 Vicryl sutures. The animals were treated with 0.05 mg/kg buprenorphine subcutaneously three times daily, or orally two times daily, the following 72 hours.

### 2.8. Bioluminescence

D-Luciferin (SynChem, Germany) was injected intraperitoneally in a dose of 30 mg/kg at days 1, 3, 7, and 14 days after the MI induction and treatment. In addition, subsets of rats were scanned at day 21. Images were acquired by IVIS® Lumina XR (Caliper Lifesciences, PerkinElmer, USA) and Living Image® software v.4.3.1 (PerkinElmer, USA) with an exposure time of 3 min, with large binning and F-stop at F8. The optimal time for imaging differed according to how many cells were present in the animal. For the first week, the optimal time of acquisition was 35 min after injection, while it was 15 min after 14 days, which could be explained by the hypoxic environment and the oxygen dependent bioluminescence reaction. A region of interest of similar size was used to collect the BLI data as luminescence counts. Background from the animal was subtracted from the data prior to analysis. BLI data were analyzed as mean luminescence counts or luminescence as percentage of day 1.

### 2.9. MRI

As with PET/CT scan, MRI was performed on days 1, 14, and 28 after the MI induction. The animals were sedated as mentioned during PET, and the scans were performed using a preclinical 7 Tesla scanner (Bruker Pharmascan, Bruker Medical, Germany) with horizontal bore and a 60 mm transmitter-receiver coil. Pilot scout images (3 slices, 1 mm thickness, field of view 3.5 × 3.5 mm, repetition time (TR) 85 ms, echo time (TE) 1.5 ms) were used to validate positioning and to image the heart in 2- and 4-chamber long axis images, as well as a perpendicular short axis image. A stack of short axis cine-FLASH sequences was acquired to cover the ventricle with 1 mm thick slices and 0.5 mm between slices. Cine-FLASH sequences were gated on respiration and ECG, with a field of view of 5.0 × 5.0 cm, matrix size of 256 × 256, TR 4.8 ms, and TE 2.0 ms, bandwidth 4566.7 Hz, 150 repetitions, and flip angle of 10°. A multislice gated T1 FLASH sequence was used to visualize the area of increased late gadolinium enhancement (LGE) 20 min after IV administration of gadolinium contrast (gadobutrol, 0.1 mol/kg, Gadovist®, Bayer Scherring Pharma, Germany). The T1 sequence used was 1 mm slices with 0.5 mm gab, field of view 6 × 6 cm, matrix size 256 × 256, TR 70 ms, TE 2.8 ms, and flip angle 30°.

Images were exported as DICOM and analyzed using dedicated software (cvi42 versus 4.0.1, Circle Cardiovascular Imaging, Canada). A total of 5–7 of the short axis cine images were used for calculation of heart volumes: left ventricular ejection fraction (LVEF), end-diastolic volume (EDV), end-systolic volume (ESV), and myocardial mass. Slices of similar location in the rat heart were used for analysis between acquisition days. Systole and diastole was manually assessed from the 15–20 frames for each slice. The endocardial and epicardial contours were drawn manually on each viable slice for the systole and diastole. For calculation of infarct area, the guided automatic LGE mapping in cvi42 was used. The infarcted area was defined as deviating more than 2.5 standard deviations from the reference myocardium. The infarct area at days 14 and 28 was defined as the area which clearly differed in thickness from the regular myocardium, and infarct thickness was measured as an average length of at least five manual thickness measurements at different locations across the infarct.

### 2.10. ^82^Rubidium-PET/CT Imaging

Rats were anesthetized with 4% sevoflurane on days 1, 14, and 28 after the operation for PET and MRI imaging. PET imaging was performed using a dedicated preclinical PET/CT scanner (Siemens Inveon, USA). After the dorsal vein cannulation by a permanent 24 G catheter, the rats were maintained on 0.5–4.0% sevoflurane during the scans. The rats were placed on a water heated bed in a prone position, with respiration and ECG monitoring. Correct positioning was validated by initial CT scout image. A ^82^Rb generator (CardioGen-82, Bracco Diagnostics Inc., USA), just expired for clinical use, was equipped with a three-way valve, allowing control of the amount of infusion to the rats [24], and calibrated according to manufacturer's recommendations. The animals received approximately 30–150 MBq ^82^Rb in 1.25–1.75 ml saline solution, shortly after a 10 min list mode PET acquisition was started. The animals were injected with 1.5 ml CT contrast (Omnipaque 350 mg, GE Healthcare, Denmark) just before a CT scan was performed for anatomical reference for attenuation correction. To minimize ^82^Rb activity from the blood pool, the PET list mode data was histogrammed into two time frames: the first 45 sec and the remaining 555 sec. Subsequently, the images were reconstructed as described previously [23].

PMOD cardiac tool version 3.3 (PMOD technologies LLC, Switzerland) was used for processing and analysis of the PET data. Initially, a fusion image of the PET and CT was created to verify the placement of the heart for attenuation correction. The PET images were then reoriented into short, vertical, and horizontal long axis. The myocardial contour was determined semiautomatically, by setting marker guidance on basal and apical points of the left ventricle, followed by visual inspection and possible correction. The perfusion was quantified as local myocardial ^82^Rb activity as percentage of maximum myocardial activity. These relative values were obtained in myocardial segments according to the 17 American Heart Association (AHA) segments [23] and as activity for the whole heart. Data were compared between scans of the individual rat, before comparisons were made between groups.

### 2.11. Histology

Immediately following animal sacrifice by decapitation during anesthesia, the heart was excised, washed in saline, retrograde perfusion from the aorta was performed with saline followed by 4% paraformaldehyde. The heart was then placed in formaldehyde for 24 hours and stored in 70% ethanol before embedding in paraffin. The hearts were cut in the short axis on a microtome, and immunohistochemistry was performed using the following: CD31 (Novus Bio, NB100-2284), CD68 (Abcam, 125212), Masson's trichrome, and hematoxylin and eosin (H&E). Prior to antibody staining, antigen retrieval was performed using citrate buffer and microwave treatment. Images were obtained by an ×20 objective scanning and subsequent stitching, producing images of the entire short axis slice. Fibrosis on Masson's trichrome stainings was quantified using ImageJ (Fiji) on the entire heart. CD31 clusters in peri-infarct regions were scored from 1–4, and CD68 was counted in the whole infarct area and normalized to the counted area.

### 2.12. Statistics

For functional measurements, changes in measured parameters within groups were assessed using paired *t*-test with Bonferroni correction, while comparisons between groups were performed using univariate analysis with day 1 as covariate and Tukey as post hoc test. Retention was compared using repeated measures ANOVA with Tukey post hoc test. All statistics were performed using SPSS (IBM SPSS statistics v. 22).

## 3. Results

### 3.1. AMI Induction and Survival

A total of 97 animals were included in this study, of which 37 did not survive the AMI procedure. Other animals died or were euthanized due to anesthesia during scanning (*n* = 8) or external wound (*n* = 1). Rats weighted 266 ± 15 g at the time of the procedure, 259 ± 17 g at day 1, 273 ± 16 g at day 14, and 300 ± 16 g at day 28.

A total of 51 rats completed the entire experimental period. Not all MRI and PET images were of optimal quality, and data from some rats could not be analyzed due to this. For MR data, a total of 38 rats were available for analysis: sham (*n* = 5), control (*n* = 9), ASCs in saline (*n* = 10), and ASCs in alginate hydrogel (*n* = 13). For BLI data, a total of 38 rats were available for analysis: ASCs in saline (*n* = 18) and ASCs in alginate hydrogel (*n* = 20). For perfusion data, 19 rats were available for analysis: sham (*n* = 2), control (*n* = 6), ASCs in saline (*n* = 8), and ASCs in alginate hydrogel (*n* = 3).

### 3.2. Effect of Alginate Hydrogel on Cell Retention

**Figure 2:**
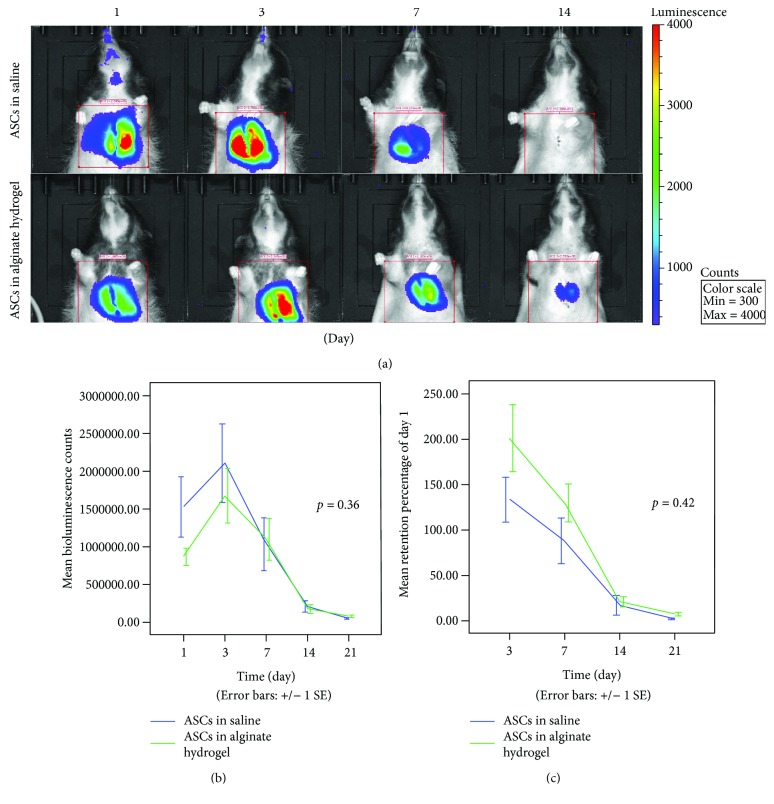
Retention of ASCs. (a) Standardized region of interest on random rats on days 1, 3, 7, and 14 of bioluminescence imaging. (b) Absolute bioluminescence count. (c) Retention of bioluminescent signal compared to day 1. Number of animals for each time point in (b) and (c): days 1, 3, and 7: *n* = 38, day 14: *n* = 24, and day 21: *n* = 14. ASC: adipose-derived stromal cells. *p* values denote differences in trajectory between the groups.

There was no significant difference in BLI signal intensity or retention between the ASC treatment groups. ASCs in saline generally produced a higher intensity BLI signal than ASCs in alginate hydrogel (*p* = 0.36). In contrast, there was a tendency for ASCs in alginate hydrogel to be better retained (*p* = 0.42) (Figure 2).

### 3.3. Improved Functional Recovery in Treatment Groups

**Figure 3:**
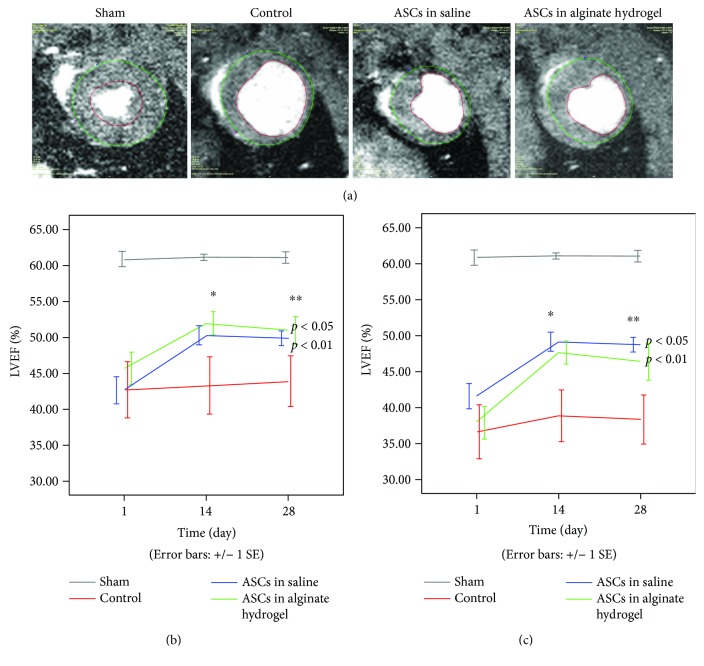
Cardiac pump function. (a) Cine image analysis of hearts from each group in systole. Endocardial (red) and epicardial (green) contours. Black arrow denotes papillary muscles in the sham group. (b) Results for left ventricular ejection fraction (LVEF) (total *n* = 38) and (c) LVEF results when excluding rats with >50% LVEF at day 1 (total *n* = 29). ∗ denotes significant difference from baseline (^∗^ *p* < 0.05; ^∗∗^ *p* < 0.01). Inserted *p* values denote difference between ASC treatment groups and control group at day 28. ASC: adipose-derived stromal cells.

LVEF increased significantly in the follow-up period in both of the treatment groups (ASCs in saline: from 42.6% to 50.3% at day 14, *p* < 0.05, and to 49.5% at day 28, *p* < 0.001; ASCs in alginate hydrogel: from 45.0% to 50.9% at day 14, *p* < 0.05, and to 50.8% at day 28, *p* < 0.001), whereas this was not the case in the control group (from 42.6% to 43.2% at day 14, NS, and to 43.9% at day 28, NS) (Table 1 and Figure 3). When comparing the change in LVEF between groups, the change was significant higher in the treatment groups compared with the control group from day 1 to day 28 (ASCs in saline versus controls: *p* < 0.01; ASCs in alginate hydrogel versus controls: *p* < 0.05), but not from day 1 to day 14 (ASCs in saline versus controls: *p* = 0.062; ASCs in alginate hydrogel versus controls: *p* = 0.087). No difference in ΔLVEF was observed between the ASC treatment groups (*p* > 0.8).

When excluding rats with smaller infarctions, defined by LVEF > 50% at day 1, the mean benefit of the treatment at day 28 was more pronounced, with ΔLVEF increased from 6.7% to 7.2% and 5.7% to 8.0% for ASCs in saline (*p* < 0.05) and alginate hydrogel (*p* < 0.01), respectively (Table 1). No significant difference was observed in ESV between the groups (Table 1), and there was no difference in LVEF or ESV between the two ASC cell lines used for treatment (*p* = 0.56).

### 3.4. Infarct Size

**Figure 4:**
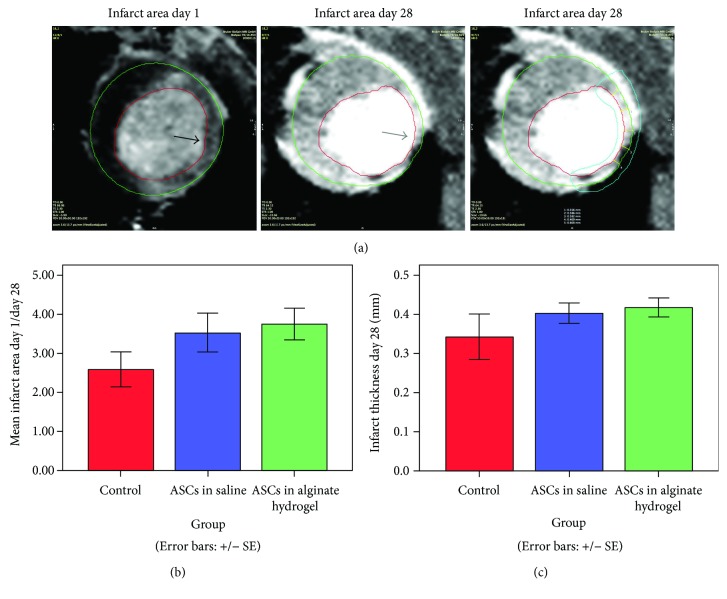
Infarct size. (a) Analysis of infarct area using representative images. Black arrow annotates gadolinium-enhanced area 15 minutes after IV injection at day 1. Grey arrow annotates clear thinning of the myocardium at day 28. The turquoise contour encircles the infarct area in the myocardium (lumen is not a part of the generated area), and the yellow lines across the defined infarct area are length measurements of infarct thickness. (b) Ratio of enhanced area at day 1 and area of myocardial thinning at day 28. (c) Infarct thickness at day 28 after infarction. For both (b) and (c), *n* = 38.

The ratio between the infarct area at day 1 and the infarct area at day 28 was used as a measure of myocardial infarct salvage. There was a tendency for greater infarct salvage in the treatment groups compared with controls, but this was not significant (ASCs in saline: *p* = 0.46; ASCs in alginate hydrogel: *p* = 0.24). The same was apparent for infarct thickness (ASCs in saline: *p* = 0.83; ASCs in alginate hydrogel: *p* = 0.58) (Figure 4).

### 3.5. Perfusion

**Figure 5:**
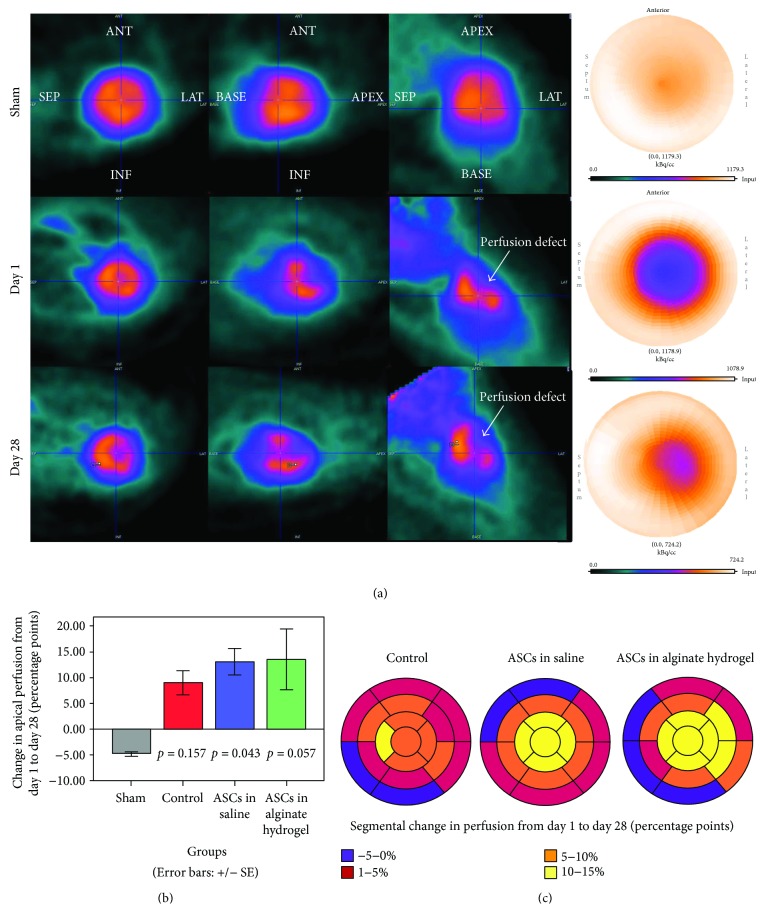
Perfusion. (a) ^82^Rb-PET imaging of rat hearts in short axis, horizontal, and vertical view, together with a bull's-eye plot of the entire left ventricle. In the upper panel is a sham heart, while a permanently ligated heart treated with ASCs in saline is shown in the middle (day 1 post MI) and lower panel (day 28 post MI). (b) Mean change in total myocardial perfusion for each group. Sample size for (b) and (c): sham = 2, control = 6, ASCs in saline = 8, and ASCs in alginate hydrogel = 3. *p* values denote difference compared to sham. (c) Mean absolute change in relative segmental perfusion in the 17 AHA heart segments. PET: positron emission tomography; ASCs: adipose-derived stromal cells; MI: myocardial infarction; AHA: American Heart Association; ANT: anterior; LAT: lateral; SEP: septal; INF: inferior.

^82^Rb-PET/CT showed that the whole heart perfusion in infarcted rats increased from day 1 to day 28 (controls and ASCs in saline: *p* < 0.05, ASCs in alginate hydrogel: *p* = 0.149). The increase in whole heart perfusion (in percentage points) at day 28 tended to be higher in the treatment groups (ASCs in saline versus controls: *p* < 0.05, ASCs in alginate hydrogel versus controls: *p* = 0.16). On a segmental basis, there tended to be an increased apical perfusion in the ASC treatment groups at day 28 compared to the sham group (ASCs in saline: *p* = 0.043, ASCs in alginate hydrogel: *p* = 0.057, controls: *p* = 0.157), but there was no significant difference between the treatment groups and controls (*p* > 0.8 for both ASC treatment groups) (Figure 5).

### 3.6. Histology

The infarct thickness measured on Masson's trichrome was controls: 655 ± 126 *μ*m, ASCs in saline: 730 ± 118 *μ*m, and ASCs in alginate hydrogel: 831 ± 404 *μ*m. There was no difference in percentage of fibrosis: controls: 33 ± 8, ASCs in saline: 36 ± 17, and ASCs in alginate hydrogel: 31 ± 8%, *p* > 0.8 for both ASC treatment groups compared with controls. More CD31-positive vessel clusters, as well as large vessels in the infarct area, were detected in the treatment groups, though not significant. There was a nonsignificant tendency for less CD68^+^ cells in the infarcted area in the treatment groups (control: 18 ± 12, ASCs in saline: 10 ± 4, *p* = 0.35 versus controls, and ASCs in alginate hydrogel: 14 ± 3 CD68^+^ cells/mm^2^, *p* = 0.63 versus controls). Sample images of each staining can be found in supplementary Figure S1.

## 4. Discussion

### 4.1. Validation of Transduced Cells

In order to increase external validation of the study, we used more than one cell line. There was no difference in efficacy or retention between the cell lines. The L2T-transduced ASC cell lines were able to triple differentiate and expressed common ASC surface markers at a level comparable to regular ASCs [25]. In addition, the cells did not show any signs of senescence, which is in line with previously results from our lab [26].

### 4.2. Retention

We did not observe an increase in cell retention using the alginate hydrogel for ASC administration, compared to injection of ASCs in saline. A large animal study with treatment of chronic infarctions also found no effect on MSC retention after two weeks using 1% alginate hydrogel as in our study, but showed improved retention using 2% alginate hydrogel [27]. Others have found increased retention using other hydrogels or formulations. Yang et al. found improved retention of syngenic transplanted Sprague-Dawley ASCs using fibrin hydrogel. They found >20% increased BLI signal compared with saline at day 14 after injection and BLI detection at day 28 only in the fibrin group [28]. Danoviz et al. showed an increased retention with both fibrin hydrogel and collagen hydrogel 24 hours after injection in rats [29].

### 4.3. Effect of Cell Treatment on Myocardial Functional Recovery

The improvements in LVEF from day 1 in the ASC treatment groups were significant already after 14 days, though not significantly increased compared to the control group, due to fewer data points at day 14 than for day 28. There is evidence that cell therapy is most efficient with large defects and lower LVEF [30–32]. Hence, we did a subgroup analysis in the animals with LVEF lower than 50% at day 1, indicating small infarctions, which resulted in more pronounced ASC treatment effect after 28 days (Table 1). The functional improvements are in line with the results from recent meta-analyses for small and large animal studies. These studies found MSC therapy for MI to improve LVEF with 7.5% in mouse AMI and ~8% in large animal MI [33–35].

### 4.4. Infarct Size and Perfusion Defect

For reports of positive results of MSC therapy for rat AMI, in addition to increased LVEF [36–40], researchers have found the treatment to increase vessel density [36, 37, 39] and scar thickness [40], while decreasing apoptosis [37, 39, 40], infarct size [36, 40], and fibrosis [36, 40]. These are usually observed using histochemistry. In line with this, we observed a tendency of increased scar thickness and decreased infarct area using MRI and histology. However, we did not observe any change in fibrosis in response to ASC treatments. This could be. Furthermore, we did observe a functional benefit of the treatment despite the lack of significant evidence of increased infarct thickness and decreased infarct size compared with controls.

The perfusion defect diminished over time after day 1 post MI and tended to diminish more in the treatment groups compared to the controls group, although not significant. This is in line with more frequent observations of clusters of vessels in the peri-infarcted area in the treatment groups (supplementary material), which has previously been observed with cell treatment for MI [41]. Perfusion for ASCs in alginate hydrogel was not significantly increased from baseline, and the change in perfusion from day 1 to day 28 was not significantly higher than sham. This is due to the small number of animals in this group (*n* = 3). This was due to technical challenges, which coincidently occurred during the scans from the alginate hydrogel group.

### 4.5. Retention and Functional Benefit

Increased retention and function have been found by Xia et al. with a thermosensitive hydrogel, which increased retention of allogeneic MSCs at 2 hours, day 1, and day 28 in a mouse AMI model. The hydrogel group exhibited increased capillary density and LVEF, as well as reduced interstitial fibrosis compared to the saline group. The increase in LVEF compared to control groups was more than doubled in the hydrogel group (~13% increase compared with ~30% increase in the saline group and hydrogel group, resp.) [42]. Similarly, Lin et al. increased retention of bone marrow mononuclear cell using self-assembling peptide nanofibers in a pig model of AMI. As with Xia et al., the functional effect of the treatment in terms of increased LVEF was more than doubled by the increase in retention [43].

The link between cell retention and functional benefit is mirrored in our study, with similar treatment effect and retention in the ASC treatment groups. The fact that the alginate hydrogel did not significantly increase ASC retention could be because it is designed for intracoronary injection following clinical AMI and not intramyocardial administration [44].

## 5. Limitations

The fact that the ASCs were injected immediately after AMI means that there is no functional measurement after the AMI and before the treatment. If there was an effect of the treatment already after 24 hours, the results of functional benefit of the treatment would therefore be blunted.

We did not include a group with alginate hydrogel without ASCs. There could be a functional effect of applying the alginate hydrogel without ASCs, but we did not investigate this, since we were mainly interested in the potential retention and function of the ASCs. Our results do not suggest an independent effect of the alginate hydrogel applied by direct intramyocardial injection, though it has been shown to protect against dilated cardiomyopathy, when applied via the coronary arteries in a pig model of AMI [44].

The infarctions were not homogeneous, resulting in large variations within the groups. This could obscure the final results. In an attempt to both show the full picture and decrease the intragroup variation, we have presented results with all data included, as well as data corrected for LVEF above 50% at day 1.

The first measurement of retention was for most animals 24 hours after administration. This is likely may indicate that we are only measuring a fraction of the administered cells, since only 10% of injected cells are retained 1 hour after intramuscular injection [7]. However, other researchers have found differences in retention using 24 hours as the first BLI time point and been able to correlate it with differences in functional heart improvement [9, 28].

The ^82^Rb-PET/CT perfusion imaging is technically challenging in small animal models, due to the combination of high positron range of the isotope and small dimensions of the heart. In addition, imaging was only performed at rest, providing data on the perfused area but not coronary flow reserve. However, this method has been shown to correlate with wall motion measured by MRI in a rat AMI model [24].

## 6. Conclusion

This study investigated for the first time if administration of human ASCs in a clinically translatable 1% alginate hydrogel could increase the retention of the injected cells and if this would affect the regenerative effect of the treatment in a rat AMI model. We found no significant effect on retention of the coadministration. Administration of human ASCs in alginate hydrogel increased pump function significantly when compared to nontreated AMI, but only to a level comparable to ASC treatment without alginate hydrogel.

## Figures and Tables

**Table 1:** Treatment effect on left ventricular (LV) function by cardiac MRI.

Group	Sham	Control	ASCs in saline	ASCs in alginate hydrogel
LV function (LVEF and LVESV) by cardiac MRI				
*LVEF* (%)				
Day 1	60.84 ± 2.21	42.64 ± 11.66	42.60 ± 5.98	45.04 ± 9.08
Day 14	61.12 ± 0.94	43.24 ± 11.05	50.32 ± 4.34	50.91 ± 4.76
Day 28	61.08 ± 1.83	43.85 ± 10.68	49.48 ± 3.75	50.75 ± 7.28
ΔLVEF 14 days	0.28 ± 1.80	1.73 ± 4.90	6.99 ± 6.26	4.81 ± 4.89
ΔLVEF 28 days	0.24 ± 1.97	1.21 ± 3.77	6.89 ± 4.67	5.71 ± 4.48
*LVESV* (ml)				
Day 1	0.12 ± 0.02	0.19 ± 0.04	0.17 ± 0.04	0.17 ± 0.05
Day 14	0.14 ± 0.04	0.23 ± 0.07	0.21 ± 0.06	0.17 ± 0.02
Day 28	0.16 ± 0.04	0.25 ± 0.07	0.22 ± 0.06	0.20 ± 0.05
ΔLVESV 14 days	0.02 ± 0.02	0.04 ± 0.04	0.05 ± 0.04	0.01 ± 0.02
ΔLVESV 28 days	0.04 ± 0.03	0.06 ± 0.05	0.05 ± 0.04	0.03 ± 0.02
*Number of animals*	*n* = 5	*n* = 9	*n* = 10	*n* = 13
Data without LVEF > 50% at day 1 in infarct groups				
*LVEF* (%)				
Day 1	60.84 ± 2.21	36.62 ± 9.17	41.57 ± 5.32	41.07 ± 8.09
Day 14	61.12 ± 0.94	38.83 ± 8.80	49.13 ± 3.28	48.64 ± 4.02
Day 28	61.08 ± 1.83	38.34 ± 8.43	48.72 ± 3.06	49.08 ± 8.13
ΔLVEF 14 days	0.28 ± 1.80	2.21 ± 5.70	7.22 ± 6.83	7.79 ± 2.91
ΔLVEF 28 days	0.24 ± 1.97	1.71 ± 4.59	7.15 ± 4.87	8.02 ± 2.30
*LVESV* (ml)				
Day 1	0.12 ± 0.02	0.21 ± 0.03	0.18 ± 0.04	0.19 ± 0.04
Day 14	0.14 ± 0.04	0.25 ± 0.03	0.22 ± 0.06	0.18 ± 0.02
Day 28	0.16 ± 0.04	0.28 ± 0.07	0.23 ± 0.05	0.22 ± 0.06
ΔLVESV 14 days	0.02 ± 0.02	0.04 ± 0.05	0.05 ± 0.05	0.00 ± 0.02
ΔLVESV 28 days	0.04 ± 0.03	0.07 ± 0.05	0.05 ± 0.04	0.03 ± 0.02
*Number of animals*	*n* = 5	*n* = 6	*n* = 9	*n* = 9

ASC: adipose-derived stromal cells; LVEF: left ventricular ejection fraction; LVESV: left ventricular end-systolic volume.

## Supplementary Materials

Supplementary Materials Images of histological stainings are provided in a supplementary figure. A) CD31-positive vessels. B) CD68-positive macrophages. C) Masson's trichrome. D) Mask of Masson's trichrome for analysis of fibrosis.
