# Supplementary material for: Retention and Functional Effect of Adipose-Derived Stromal Cells Administered in Alginate Hydrogel in a Rat Model of Acute Myocardial Infarction
Source: Stem Cells Int. 2018 Mar 26;2018:7821461. doi: 10.1155/2018/7821461 (PMC5892231; doi:10.1155/2018/7821461)
Supplement: Supplementary Materials — Images of histological stainings are provided in a supplementary figure. A) CD31-positive vessels. B) CD68-positive macrophages. C) Masson's trichrome. D) Mask of Masson's trichrome for analysis of fibrosis. [file 7821461.f1.pptx]

## Slide 1
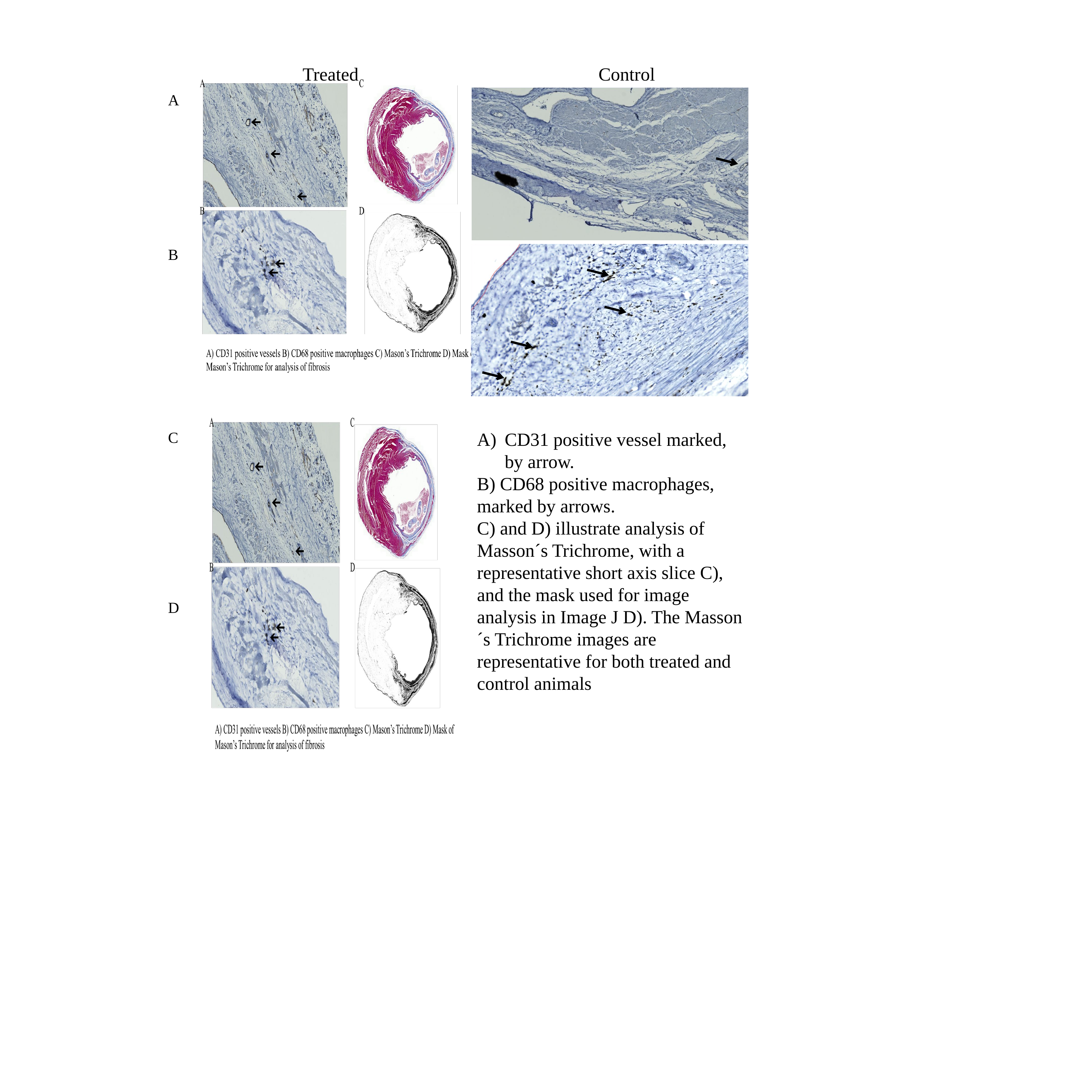

Treated
Control
CD31 positive vessel marked, by arrow.
B) CD68 positive macrophages, marked by arrows.
C) and D) illustrate analysis of Masson´s Trichrome, with a representative short axis slice C), and the mask used for image analysis in Image J D). The Masson´s Trichrome images are representative for both treated and control animals
A
B
C
D
